# DNase I Induces Other Endonucleases in Kidney Tubular Epithelial Cells by Its DNA-Degrading Activity

**DOI:** 10.3390/ijms21228665

**Published:** 2020-11-17

**Authors:** Tariq Fahmi, Xiaoying Wang, Dmitry D. Zhdanov, Intisar Islam, Eugene O. Apostolov, Alena V. Savenka, Alexei G. Basnakian

**Affiliations:** 1 Department of Pharmacology & Toxicology, University of Arkansas for Medical Sciences, 4301 West Markham Street, #638, Little Rock, AR 72205, USA; Tariq.Fahmi@fda.hhs.gov (T.F.); shawingw@hotmail.com (X.W.); zhdanovdd@gmail.com (D.D.Z.); SKhan4@uams.edu (I.I.); apostolove1@gmail.com (E.O.A.); SavenkaAlenaV@uams.edu (A.V.S.); 2 Central Arkansas Veterans Healthcare System, 4300 West 7th Street, Little Rock, AR 72205, USA

**Keywords:** DNase I, endonucleases, DNA breaks, cell death, apoptosis

## Abstract

Endonuclease-mediated DNA fragmentation is both an immediate cause and a result of apoptosis and of all other types of irreversible cell death after injury. It is produced by nine enzymes including DNase I, DNase 2, their homologs, caspase-activated DNase (CAD) and endonuclease G (EndoG). The endonucleases act simultaneously during cell death; however, regulatory links between these enzymes have not been established. We hypothesized that DNase I, the most abundant of endonucleases, may regulate other endonucleases. To test this hypothesis, rat kidney tubular epithelial NRK-52E cells were transfected with the DNase I gene or its inactive mutant in a pECFP expression vector, while control cells were transfected with the empty vector. mRNA expression of all nine endonucleases was studied using real-time RT-PCR; DNA strand breaks in endonuclease genes were determined by PCR and protein expression of the enzymes was measured by Western blotting and quantitative immunocytochemistry. Our data showed that DNase I, but not its inactive mutant, induces all other endonucleases at varying time periods after transfection, causes DNA breaks in endonuclease genes, and elevates protein expression of several endonucleases. This is the first evidence that endonucleases seem to be induced by the DNA-degrading activity of DNase I.

## 1. Introduction

Apoptotic endonucleases are a group of endonucleases (DNases or DNase/RNases), which are important as DNA destroyers for cell death [1,2]. The term “apoptotic” is traditionally used, but is not exactly correct because these enzymes are active in all kinds of cell death [3]. Organs of the digestive system and kidney are known for their high activity of apoptotic endonucleases [4,5,6,7], where the enzymes are cytotoxic themselves or promote the cytotoxicity of other agents [1,8,9,10]. There are nine known apoptotic endonucleases [3], including deoxyribonuclease 1 (DNase I) [7,11], deoxyribonuclease 2 (DNase 2) [12], their homologs [13,14,15], caspase-activated DNase (CAD) [10,16], and endonuclease G (EndoG) [17,18,19]. Induction and nuclear import of the endonucleases is attributed to an irreversible stage of cell death manifested with the fragmentation of DNA [3,9,11].

Recent studies showed that inactivation of apoptotic endonucleases in the kidney or other organs prior to cell injury protects them against cell death, suggesting that DNA fragmentation is an important mediator of cell death [4,5,9,20]. Some of these apoptotic endonucleases have been known for many years [21,22,23]; however, studying their specific roles in cell death is problematic for many reasons. For example, the same enzymes remain active in live cells and are responsible for DNA disposal after cell death [1,8,9], which makes it difficult to study the roles of these enzymes in live cells. Specific chemical inhibitors of these endonucleases suitable for in vivo use and are still under development. Genetic knockouts of these endonucleases often lack specific phenotypes [4,24,25,26]. All endonucleases catalyze the same reaction of destroying DNA by hydrolysis of phosphodiester bonds [1,8,9]. Endonuclease specificity to primary DNA structure is sometimes observed at the initial stages of DNA fragmentation, but it usually disappears quickly and is not present at later stages. All the enzymes share a role in cell death and DNA disposal which is present in all cells and tissues, and the endonucleases are often activated simultaneously in the same cell [1,27].

Other cell death enzymes, such as caspases and protein kinases, are known to act in networks by activating each other [28,29]. However, it remains to be established whether endonucleases are mechanistically linked with each other, or whether there is a network of endonucleases acting in a cooperative manner. Our previous study demonstrated that EndoG is capable of inducing other endonucleases [30]. It seemed logical to propose that DNase I, which is the most studied [4,5,7,11,20,21,31], the most abundant [7], and the most active apoptotic endonuclease [4] would also induce other endonucleases. In this study, we evaluated whether the DNA-degrading activity of DNase I is the underlying mechanism of other endonucleases’ induction in kidney tubular epithelial cells.

## 2. Results

### 2.1. Generation of Inactive DNase I Mutant

**Figure 1:**
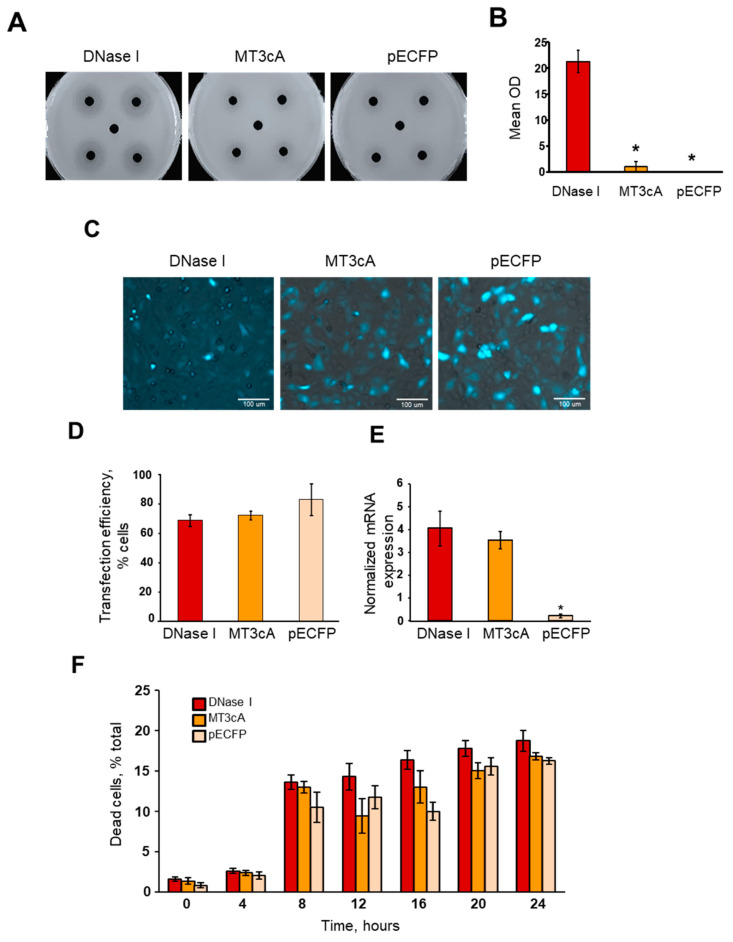
Overexpression of native DNase I or inactive DNase I mutant MT3cA in kidney tubular epithelial cells. (**A**) The DNase activity in the total protein extract of NRK-52E cells 24 h post transfection was measured using an SRED assay. Representative images showing DNA degradation circles in agar gel caused by pECFP-DNase I, pECFP-MT3cA or control pECFP transfection. (**B**) Quantification of the SRED assay data (*n* = 4, * *p* < 0.01 compared to DNase I). (**C**) Representative fluorescent/phase contrast (CFP + PhC) images showing NRK-52E cells transfected with pECFP-DNase I, pECFP-MT3cA, or pECFP 8 h after transfection. (**D**) Total fluorescence of the transfected cells 8 h after transfection. (**E**) DNase I expression in NRK52E cells transfected with pECFP-DNase I, pECFP-MT3cA (its inactive mutant) or pECFP measured by real-time RT-PCR 8 h after transfection. mRNA expression levels were normalized by 18 s housekeeping gene (*n* = 4). (**F**) PI cell death assay showing the mean percentage of dead NRK-52E cells transfected with pECFP-DNase I, pECFP-MT3cA, or pECFP 0-24 h after transfection (*n* = 4, * *p* < 0.01 compared to DNase I). In panel (**F**), the difference in cell death was not statistically significant between the constructs.

Inactive DNase I mutant, MT3cA, was produced as described in Materials and Methods. Kidney tubular epithelial cells were chosen because they are one of the primary sites of endonuclease expression in the body [4,32,33]. To determine whether the inactivation was complete, NRK-52E cells were transfected with a vector that encoded either native DNase I (DNase I) gene or mutated MT3cA DNase I, both of which were fused with CFP. Control cells were treated with an “empty” pECFP vector that encoded CFP protein. The total protein was extracted from transfected cells, and DNase I activity was assessed using an SRED assay. This method showed significant DNase activity in cells transfected with pECFP-DNase I (Figure 1). DNase I activity of cells transfected with pECFP-MT3cA was indistinguishable from activity in cells transfected with control pECFP indicating that the inactivation of all seven DNA binding sites in the DNase I molecule caused almost complete inactivation of its DNA-degrading activity.

### 2.2. Validation of DNase I or MT3cA Overexpression in Renal Tubular Epithelial Cells

DNase I was previously shown to induce apoptosis after being overexpressed in mammalian cells [11]. Therefore, we first tested whether or not DNase I overexpression would induce cytotoxicity, which was determined by assessment of cell number, measurement of cell death and yields of total cellular DNA or RNA. NRK-52E cells were transfected with pECFP-DNase I, pECFP-MT3cA, or pECFP control plasmid. Based on image quantification, the transfection efficiency was not different between cells transfected with the three constructs in a period of time from 0 to 24 h after transfection (Figure 1C,D). The analysis of the total DNA and RNA isolated from the transfected cells demonstrated a sufficient yield and an equally good quality of nucleic acids at every studied time point in a period of 24 h with no difference in respect to DNase I, MT3cA and control pECFP overexpression (data not shown). Real-time RT-PCR demonstrated no difference in the expression of DNase I in NRK-52E cells transfected with pECFP-DNase I or pECFP-MT3cA; however, both were significantly higher than DNase I expression in cells transfected with pECFP (Figure 1E). The PI cell death assay showed a time-dependent cytotoxicity of all three constructs used for transfection. Overexpression of active DNase I tended to generate on average a higher cytotoxicity than pECFP or MT3cA, however the difference did not reach the level of statistical significance (Figure 1F). These data indicate that at the used levels of overexpression, the cells were not significantly affected by the transfection to cause spontaneous cell death.

### 2.3. DNase I Overexpression Induces Other Endonucleases

**Figure 2:**
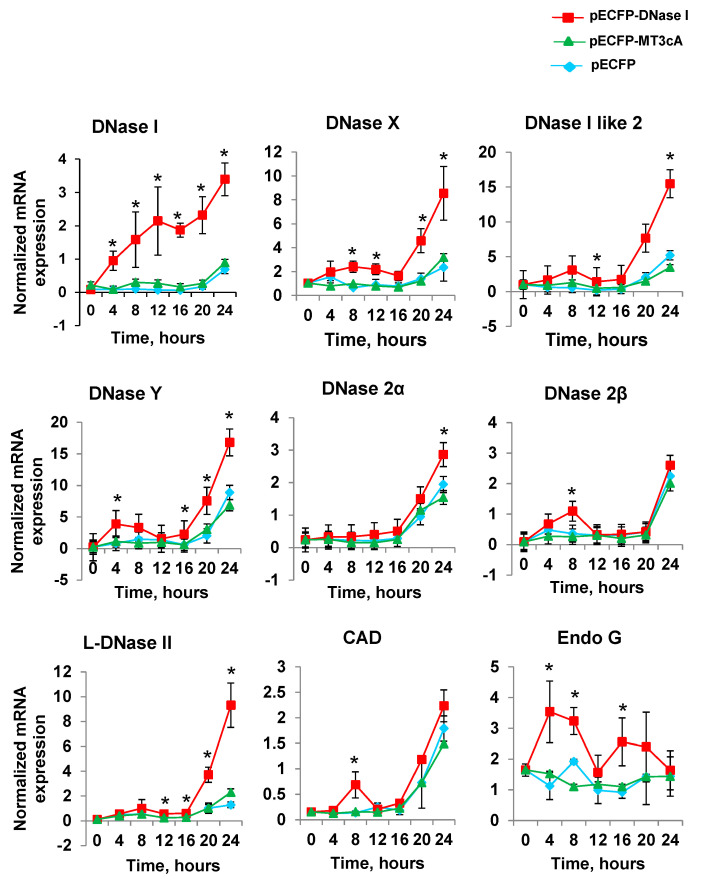
DNase I overexpression causes induction of other endonucleases. NRK-52E cells were transfected with pECFP, pECFP-DNase I or pECFP-MT3cA and mRNA expressions of endonucleases were measured by real-time RT-PCR with 4 h intervals. mRNA expression levels were normalized by 18 s housekeeping gene (*n* = 4, * *p* < 0.05 compared to pECFP and pECFP-MT3cA).

To determine whether the expression of any known apoptotic endonucleases is affected by the overexpression of DNase I, real-time RT-PCR was performed in both pECFP-DNase I-, pECFP-MT3cA- and pECFP-transfected cells every 4 h within 24 h after transfection (Figure 2). DNase I expression was significantly increased in pECFP-DNase I-transfected cells compared to those transfected with pECFP at all time points. Two of the studied endonucleases, EndoG and DNase γ, were induced by DNase I as early as 4 h post transfection. DNase X, DNase I like 2, DNase 2β and CAD were induced at 8 h. L-DNase 2 and DNase 2α were induced at 12 h after transfection. Therefore, all other endonucleases were induced by DNase I overexpression within 4 to 24 h post transfection.

### 2.4. Overexpression of Inactive DNase I Mutant Does Not Induce Other Endonucleases

**Figure 3:**
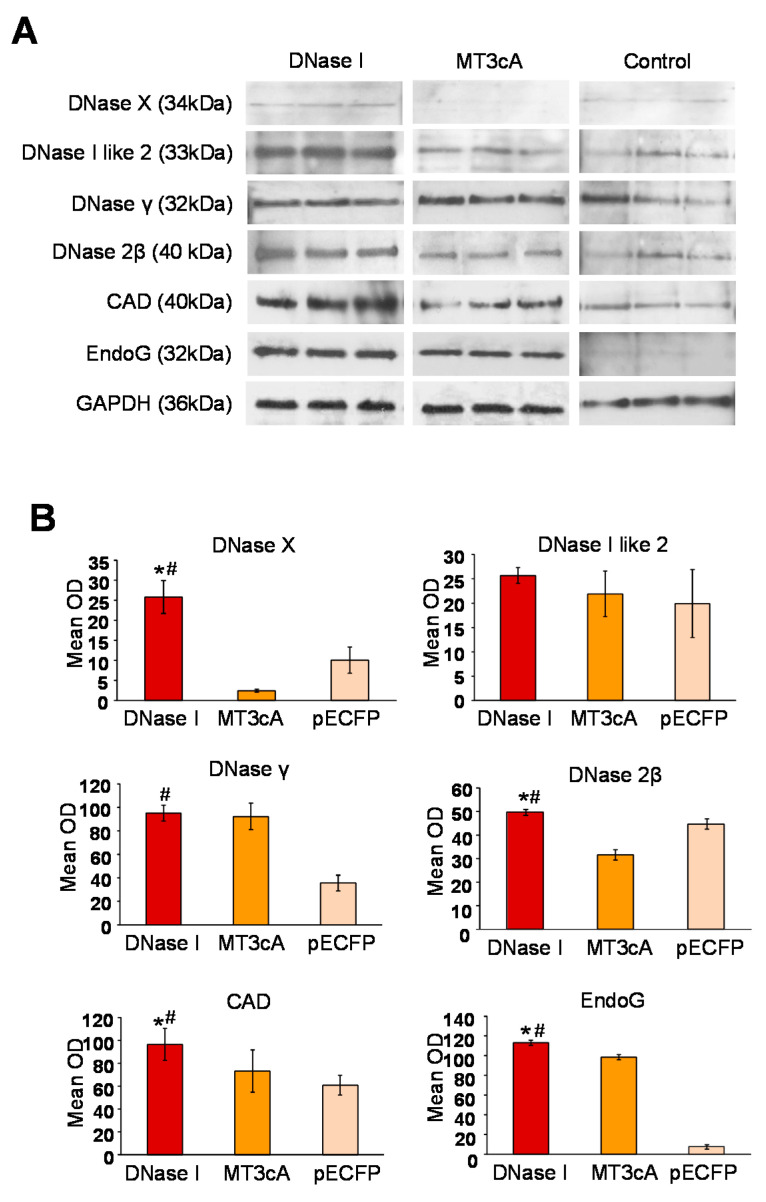
Inactive DNase I mutant does not induce other endonucleases. (**A**) Western blotting for DNase X, DNase I like 2, DNase γ, DNase 2β, CAD, EndoG, and GAPDH (as a loading control) in NRK-52E cells 24 h after transfection with pECFP-DNase I or pECFP-MT3cA. (**B**) Quantification of DNase X, DNase I like 2, DNase γ, DNase 2β, CAD, and EndoG by densitometric analysis of band mean optical densities (* *p* < 0.05 compared to pECFP-MT3cA; # *p* < 0.05 compared to pECFP-transfected NRK-52E cells).

To determine whether the inactivation of DNA-degrading activity of DNase I would change or abolish the induction of other endonucleases, we compared the effect of DNase I with that of its inactive mutant, MT3cA in the next experiment. A single time point of 8 h after transfection was chosen because at this time, as shown above, several endonucleases were induced by DNase I. Real-time RT-PCR demonstrated a significant decrease in the expression of all endonucleases in NRK-52E cells transfected with pECFP-MT3cA compared to pECFP-DNase I (Figure 2). To evaluate the protein expression of individual endonucleases, total protein was extracted from NRK-52E cells 24 h after transfection with pECFP-DNase I or pECFP-MT3cA. Western blotting was performed with all available antibodies to endonucleases (Figure 3A). Quantification of the Western blotting data showed that DNase I overexpression significantly increased expression of all endonucleases with the exception of DNase I like 2 (Figure 3B). Transfection with pECFP-MT3cA did not induce expression of DNase X, DNase 2β, CAD, EndoG, and DNase γ. Similarly to pECFP-DNase I, pECFP-MT3cA did not change the expression of DNase I like 2. Taken together, these data indicate that the inactivation of DNA-degrading activity of DNase I abolishes its ability to induce several other cellular endonucleases. In other words, DNA breaks are the potential mechanism of other endonucleases’ induction by DNase I.

**Figure 4:**
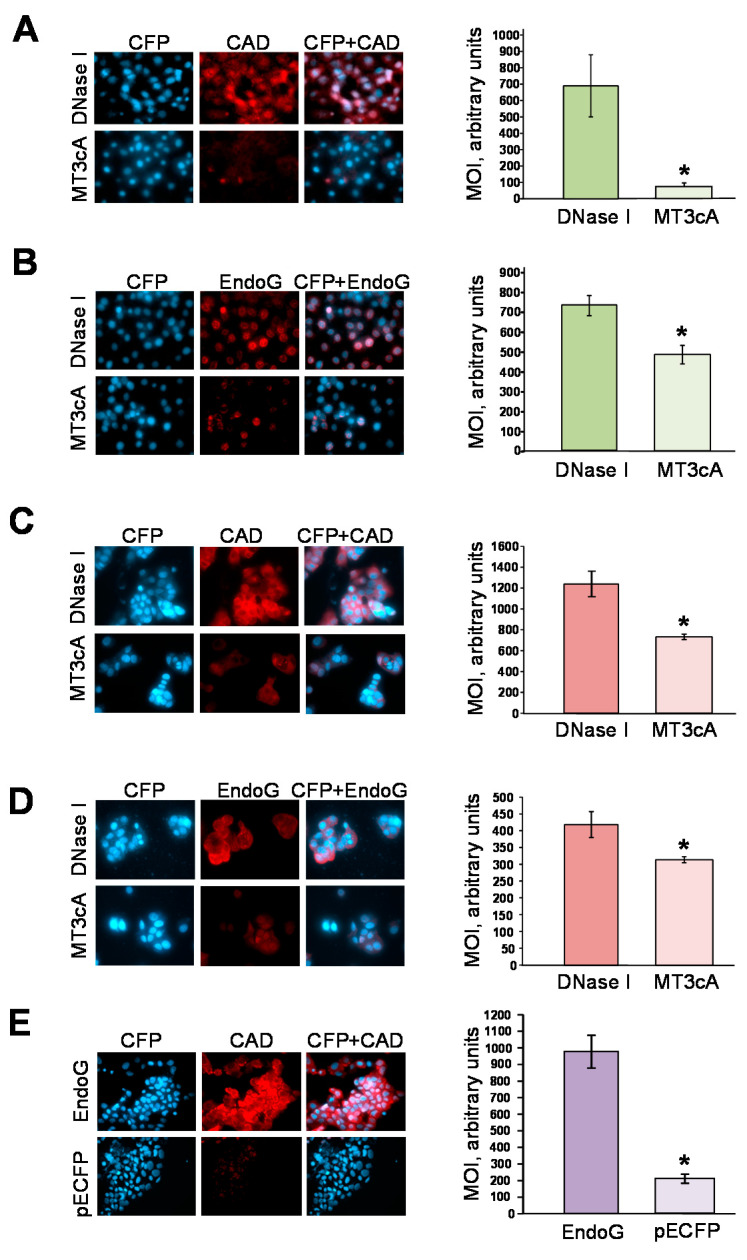
Inactive DNase I mutant does not induce EndoG or CAD proteins, while EndoG induces CAD. (**A**) CAD immunostaining and quantification of mean optical density (MOD) in NRK-52E cells 8 h after transfection with pECFP-DNase I or pECFP-MT3cA (* *p* < 0.01 compared to pECFP-MT3cA). (**B**) EndoG immunostaining and quantification of MOD in NRK-52E cells 4 h after transfection with pECFP-DNase I or pECFP-MT3cA (* *p* < 0.05 compared to pECFP-MT3cA). (**C**) CAD immunostaining and quantification of MOD in ZR-75-1 cells 8 h after transfection with pECFP-DNase I or pECFP-MT3cA (* *p* < 0.01 compared to pECFP-MT3cA). (**D**) EndoG immunostaining and quantification of MOD in ZR-75-1 cells 4 h after transfection with pECFP-DNase I or pECFP-MT3cA (* *p* < 0.05 compared to pECFP-MT3cA). (**E**) CAD immunostaining and quantification of MOD in NRK-52E cells transfected with pECFP-EndoG or pECFP 4 h after transfection (* *p* < 0.01 compared to pECFP).

In another approach, to confirm that the protein expression of other endonucleases is not induced by MT3cA, we used quantitative immunocytochemistry to measure the protein expression of CAD and EndoG, two endonucleases which play the most active role in apoptotic DNA fragmentation and cell death [10,17,18]. The amount of CAD and EndoG proteins in cells transfected with pECFP-MT3cA was significantly less than in cells transfected with pECFP-DNase I (Figure 4A,B). This additionally confirmed that the apoptotic endonucleases are induced by DNase I-generated DNA breaks. In parallel, we transfected another cell line ZR-75-1, which is known to have lower basal level of DNase I expression [34] with either pECFP-DNase I or pECFP-MT3cA followed by immunostaining for CAD and EndoG. Quantification showed a significant increase in the expression of both CAD and EndoG in the cells transfected with pECFP-DNase I compared to pECFP-MT3cA-transfected cells (Figure 4C,D). Another experiment was performed to determine whether overexpression of EndoG instead of DNase I would have a similar effect. NRK-52E cells were transfected with pECP-EndoG and pECFP (control) and immunostained for CAD. Quantitative immunocytochemistry showed a significant increase in CAD expression in the cells transfected with EndoG compared to pECFP-transfected cells (Figure 4E). Therefore, it seems that the nature of DNA breaks may not matter for all endonucleases induction: they may come from one or another endonuclease.

### 2.5. DNase I Overexpression Induces Degradation of Endonuclease Genes

**Figure 5:**
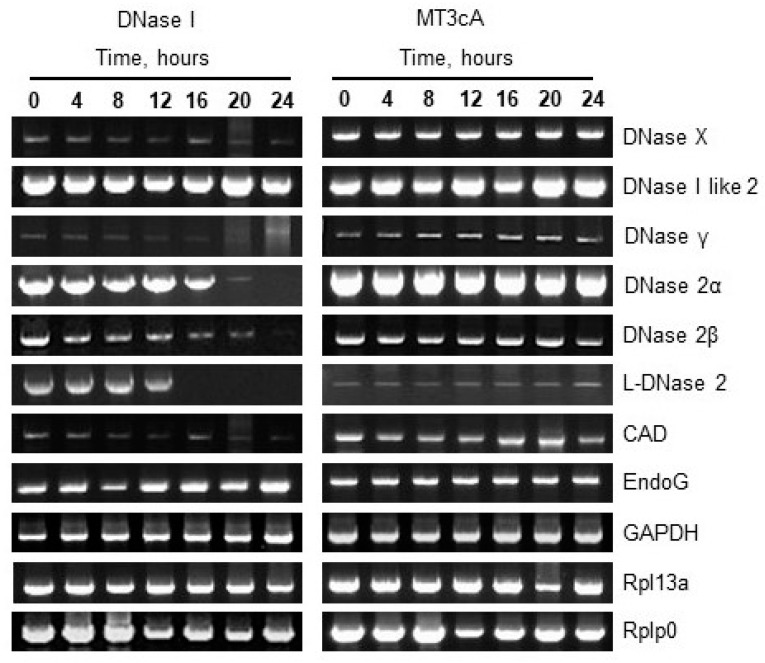
DNase I overexpression induces breaks in the promoter/exon 1 regions of endonucleases genes. PCRs of genomic DNA in promoter/exon 1 regions of endonucleases and housekeeping genes (GAPDH, PPL13a, and Rplp0) were performed using primers listed in Table S1.

We then tested whether DNase I overexpression would induce DNA breaks in endonuclease genes, and if this would coincide with the induction of endonuclease mRNAs. NRK-52E cells were transfected with pECFP-DNase I or pECFP-MT3cA and PCR was performed with DNA every 4 h for up to 24 h post transfection. The PCR for endonuclease genes was performed in the promoter/exon 1 areas, which are likely to be involved in regulation of the genes. To allow comparison, the PCR was designed to generate products of approximately the same length or 3 kb. The results presented in Figure 5 show some DNA breaks (at least one double-stranded or two single-stranded DNA breaks per 3 kb) in the promoter/exon 1 region in most of the endonuclease genes. This occurred rather late after transfection. In particular, the genes of DNase X, DNase γ, DNase 2α, DNase 2β, L-DNase 2, and CAD got these DNA breaks at varying time points between 12 and 24 h post transfection. The only two genes which showed no DNA breaks were DNase I like 2 and EndoG. Importantly, the degradation was completely abolished when MT3cA mutant was used instead of DNase I. Therefore, the degradation of endonuclease genes was also dependent on the DNA-degrading activity of DNase I.

### 2.6. Recombinant DNase I Induces Other Endonucleases

**Figure 6:**
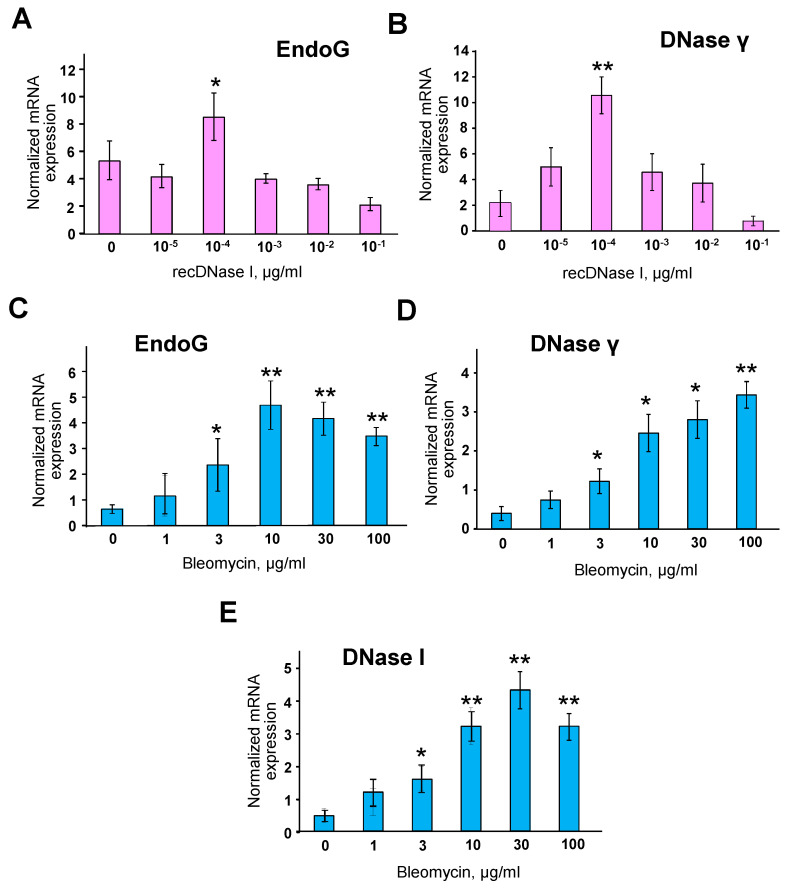
Both DNase I and bleomycin induce EndoG and DNase γ transcription in isolated rat brain cell nuclei, while bleomycin also induces DNase I transcription. In vitro transcription of EndoG (**A**) and DNase γ (**B**) genes in isolated rat brain nuclei exposed with varying concentrations of recombinant DNase I (Pulmozyme) as measured by real-time RT-PCR (*n* = 4, * *p* < 0.05, ** *p* < 0.01 compared to 0 concentration). (**C**–**E**) In vitro transcription measured by real-time RT-PCR of EndoG, DNase γ and DNase I genes, respectively, in isolated rat brain nuclei exposed with varying concentrations of bleomycin. mRNA expression levels were normalized by 18 s housekeeping gene (*n* = 4, * *p* < 0.05, ** *p* < 0.01 compared to 0 concentration).

To determine whether DNase I can directly induce the expression of other endonucleases by acting on chromatin, we used rat brain nuclei, which are notorious for their very low endogenous endonuclease activity [35]. The cell nuclei were isolated and exposed with varying concentrations of recombinant DNase I for 20 min. Total RNA was extracted and real-time RT-PCR was performed for EndoG and DNase γ mRNAs, the two endonucleases prominently induced by overexpression of DNase I as shown above. Both of the mRNAs were significantly induced at 0.1 ng/mL DNase I (Figure 6A,B). As expected, higher concentrations of DNase I reduced the expression of the endonucleases to the control levels or below, likely due to the excessive DNA degradation. To determine whether DNA breaks of non-enzymatic origin would induce endonucleases in the same manner, we tested EndoG, DNase γ, and DNase I expression in isolated rat brain nuclei treated with varying concentrations of bleomycin, a known inducer of oxidative DNA breaks [36]. Real-time RT-PCR showed a significant increase in the expression of these endonucleases at all bleomycin concentrations, which suggested that non-enzymatic DNA breaks induce the endonucleases similarly to the enzymatic breaks (Figure 6C,D).

### 2.7. DNase I Digestion Results in DNA Unwinding

**Figure 7:**
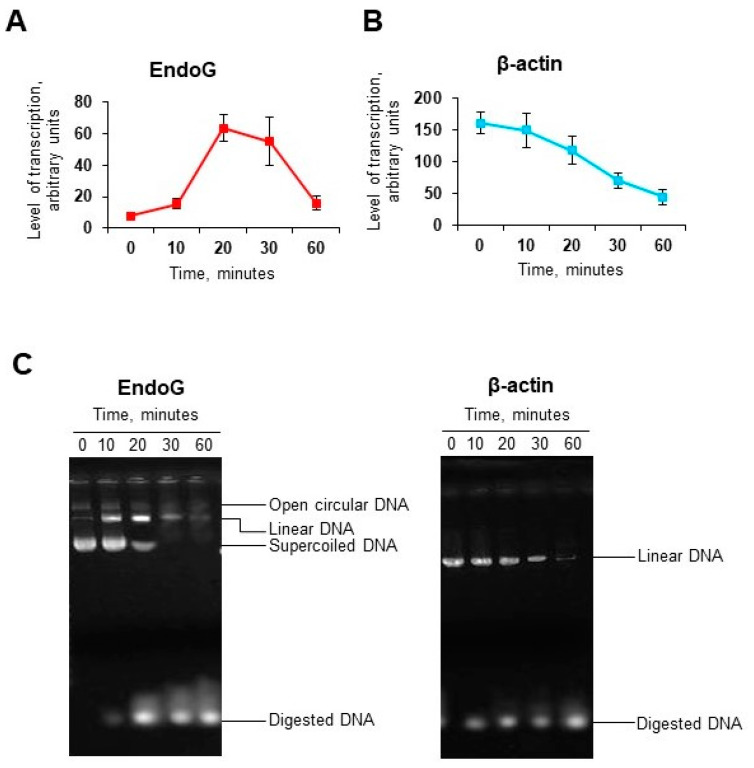
In vitro transcription of mouse EndoG (**A**) and mouse beta-actin (**B**) genes in plasmid constructs cleaved with recDNase I. mRNA expression was measured by real-time RT-PCR. Arbitrary units reflect coefficient of dilutions from 1:40 to 1:320 that were applied to the standard curve (*n* = 4). (**C**) Gel electrophoresis shows digestion of pET29b-mEndoG and pTRI-β-actin plasmids by recDNase I (Pulmozyme).

To additionally test whether DNase I can induce expression of another endonuclease, supercoiled plasmid coding mouse EndoG and linearized plasmid coding mouse beta-actin (in vitro transcription kit control) were treated with recDNase I for up to 60 min followed by in vitro transcription and real-time RT-PCR. Beta-actin mRNA levels were high at 0 time point, meaning that the transcription kit worked, and then went down to the lowest level at 60 min (Figure 7B). EndoG mRNA expression was initially low when supercoiled DNA was used, then started to increase after 10 min, peaked at 20 min, and the decreased similar to beta-actin (Figure 7A). Digested plasmids were subjected to gel electrophoresis, which showed how digestion of supercoiled plasmid results in relaxation of the plasmid and its conversion to the linear form. These data indicate that DNase I-mediated DNA breaks result in the unwinding of DNA causing the increased transcription of EndoG gene.

### 2.8. Increased Expression of DNase I and Other Endonucleases, Except EndoG, Correlate with Cell Death

The expressions of both DNase I and CAD measured by real-time RT-PCR highly correlated with cell death measured by PI assay. This is also true for other enzymes including DNase 2α, DNase I like 2, L-DNase II, DNase γ, DNase X, and DNase 2β. Surprisingly, EndoG was the only endonuclease whose expression did not correlate with cell death (Figure S1). EndoG is unique among the apoptotic endonucleases tested here in that it also has RNase activity which may affect the role of EndoG in cell death apart from its DNase activity.

## 3. Discussion

This study is the first demonstration of a previously unknown ability of DNase I to induce other apoptotic endonucleases via DNA breaks. This group of endonucleases includes nine enzymes: four members of the DNase I family, three members of the DNase 2 family, CAD and EndoG [3,7,10,11,12,13,14,15,17,18]. The endonucleases can act independently by catalyzing the same reaction of hydrolysis of phosphodiester bonds immediately prior, during, and after cell death. However, regulatory links between the endonucleases have not previously been established. We hypothesized that DNase I, the most active apoptotic endonuclease, may regulate other endonucleases. To test this hypothesis, rat kidney tubular epithelial NRK-52E cells were transfected with rat Dnase I gene or its inactive mutant MT3cA in a pECFP expression vector, while control cells were transfected with the empty pECFP vector. We found that DNase I, but not its inactive mutant, induces all other endonucleases at varying time periods after transfection. DNase I overexpression also induced DNA breaks in the promoter/exon 1 regions of endonuclease genes, and protein expression of several of the endonucleases. In this transfection model, we showed that the entire group of endonucleases was regulated by the DNA-degrading activity of DNase I. Our experiments with isolated cell nuclei treated with recombinant DNase I suggest that the likely mechanism is a direct DNA unwinding by the DNase, making DNA accessible for transcription. Unexpectedly, we found that DNA degradation with the intensity of at least one double-stranded or two single-stranded DNA breaks per 3 kb induced by DNase I in the promoter/exon 1 regions of other endonuclease genes does not completely suppress RNA synthesis. Apparently, some endonuclease mRNAs continued to be synthesized at even higher rates after DNase I treatment despite the apparent start of DNA template destruction.

**Figure 8:**
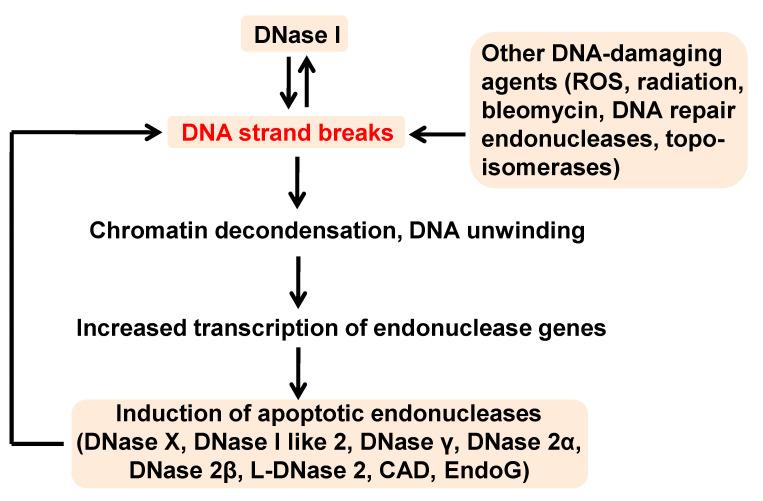
Hypothetical scheme of apoptotic endonuclease network.

This study is in agreement with our previous report that showed EndoG is able to induce other endonucleases [30]. It is also in agreement with the report from Yin et al. [5], which showed that the induction of EndoG expression in kidneys during cisplatin injury depends on the expression of DNase I. In another study, a DNase I inhibitor and the genetic inactivation of DNase I in mice regulated DNase γ endonuclease activity in the spleen [37,38]. After being simultaneously activated, DNase I and EndoG are likely to cooperate in the generation of double-stranded DNA cleavage products as described by Widlak et al. [39] DNase I was also shown to cooperate with DNase gamma and CAD [27]. Taken together, these data suggest the existence of a potential endonuclease network, which unite all apoptotic endonucleases as well as other enzymatic and non-enzymatic mechanisms of DNA fragmentation (Figure 8). Since we observed DNase I acting through DNA breaks, we hypothesize that other DNA-breaking enzymes (for example, other apoptotic or DNA repair endonucleases or topoisomerases), reactive oxygen species (ROS), or physical and chemical agents (such as radiation or bleomycin) acting through DNA breaks would have the same effect of inducing apoptotic endonucleases. Therefore, DNase I is the upstream and downstream participant of the endonuclease network. It contributes to the pool of DNA breaks, and together with other endonucleases, seems to induced by the DNA breaks.

There are two other enzyme networks known to associate with cell death, caspases and protein kinases [28,29]. There appears to be a common mechanism of self-promoting enzyme networks in cells which continue to work even during cellular destruction. The resiliency of this mechanism during events where cellular systems are being disintegrated highlights the importance of this enzyme-based cell-death system. Each of these networks is based on the activity of its members, proteinases or kinases, and thus is capable of acting independently of the normal regulatory transcriptional pathways. In this regard, the endonuclease network seems to work similarly.

Future studies may need to determine whether other endonucleases would work similarly to DNase I, and if any and all non-enzymatic DNA breaks can induce endonucleases. It seems likely that the mediation of DNase I action through DNA breaks is a universal mechanism. Other endonucleases also can produce DNA breaks, or DNA breaks can be induced by physical and chemical agents. All of these would be expected to induce endonucleases. The important consequence of this finding may be that inhibiting a single endonuclease for tissue protection against injury is likely to be less efficient than inhibiting several endonucleases and other DNA-degrading activities at once.

Our data indicate that DNase I acts as some kind of a transcription factor. This is similar to the activation of Fas transcription after internalization of extracellular DNase I reported by Oliveri et al. [40]. Although DNase I DNA-cutting activity is important for induction of other endonucleases, it is yet to be determined whether DNase I acts alone while fragmenting DNA, or if it employs other endonucleases to do this as well. Current methods do not distinguish between DNA breaks induced by DNase I and other endonucleases. Therefore, in light of our data, previously observed apoptotic DNA fragmentation induced by overexpression of DNase I in COS cells [11] can be explained by activation of other endonucleases by DNase I. DNA breaks induced by DNase I and other endonucleases will likely induce DNA damage pathways. Activation of endonucleases at different time points after DNase I overexpression, and the existence of more than one peak (for example, for EndoG), may indicate that more than one mechanism of endonuclease induction links DNase I with other endonucleases. DNase I is not the most effective instrument to degrade DNA because it generates single-stranded breaks, which can be easily repaired. For the fastest degradation, DNA needs to be cut by double-stranded DNA breaks. It would be interesting to study whether DNase 2, which produces double-stranded DNA scissions, would induce endonucleases in the same way as DNase I. In addition, specificity of endonucleases to induce other endonucleases will require further investigation.

In summary, we found that DNase I activity at non-toxic levels induced the entire network of apoptotic/cytotoxic endonucleases. The endonuclease network seems to be an effective mechanism to ensure fast DNA degradation and cell death after a deadly stimulus. Multiple endonucleases working in a network of expression induction would provide the flexibility of responses and guarantee the effective DNA fragmentation after any stimulus. The discovery of this endonuclease network and the mechanism that mediates it may have broad implications. It provides a new perspective on mechanisms of cell-death which could guide the development of cell-preserving, anti-apoptotic therapy for various organ injuries caused by trauma, hypoxia/oxidant, radiation, drugs or diseases. Further studies may also link enzymatic DNA fragmentation, DNA repair, and non-enzymatic DNA damage in a single pathway.

## 4. Materials and Methods

### 4.1. DNase I and EndoG Cloning and Site-Direct Mutagenesis

A previously cloned rat DNase I gene [41] or rat EndoG gene was inserted in the pECFP-N1 vector (Clontech, Mountain View, CA, USA) upstream of the ECFP gene. Inactive DNase I mutant, MT3cA, with seven DNA binding sites inactivated by mutation (R9G, R41A, Y76A, N170A, R111G, Y175A, Y211A), was produced using modified QuikChange^®^ XL Site-Directed Mutagenesis Kit (Stratagene, La Jolla, CA, USA).

### 4.2. Cell Culture

Normal rat tubular epithelial NRK-52E cells (ATCC, Manassas, VA, USA) were grown in Dulbecco’s Modified Eagle’s Medium (DMEM) (ATCC) supplemented with 5% fetal bovine sera. Human breast cancer ZR-75-1 cells (ATCC) were grown RPMI-1640 with 10% fetal bovine sera. Cells were cultured at 5% CO_2_/95% air in a humidified atmosphere at 37 °C, fed at intervals of 48–72 h, and used within 1 day after confluence. The cells were transfected using Lipofectamine 3000 (Invitrogen, Grand Island, NY, USA) and the expression of fusion DNase I-cyan fluorescent protein (CFP) or CFP alone (control) was detected by fluorescent microscopy.

### 4.3. Propidium Iodide Cell Death Assay

Transfected NRK-52E cells were incubated at 37 °C with 40 μM propidium iodide (PI) for 30 min, and the fluorescence of dead cells was measured in a plate reader at 530/645 nm (reading R1). Cells were then permeabilized with 10% Triton X100 for 20 min, and the total fluorescence was measured at the same wavelength (reading R2). The percentage of dead cells was calculated as the ratio R1/R2.

### 4.4. Isolation of Cell Nuclei and In Vitro Transcription

Rat brain nuclei were isolated as previously described [35]. The nuclei were exposed with varying concentrations of human recombinant DNase I (Pulmozyme, Genentech, San Francisco, CA, USA) or bleomycin (Sigma, St. Louis, MO, USA) for 20 min in 50 mM Tris-HCl, pH 7.9, 0.25 M sucrose, 10 mM MgCl_2_, 1 mM CaCl_2_, 5 mM 2-mercaptoethanol. Total RNA was extracted and real-time RT-PCR was performed as described below. For in-vitro transcription, Mouse-EndoG/pET29b (Novagen) and linear pTRI-β-actin-Mouse contained T7 promoter (Ambion, Austin, TX, USA) were exposed to human recombinant DNase I (recDNase I) (0.1 μg/mL) (Pulmozyme, Genentech, San Francisco, CA, USA) for varying time periods, and then Maxi script T7 kit (Ambion, Austin, TX, USA) was used according to the manufacturer protocol.

### 4.5. Single Radial Enzyme Diffusion (SRED) Assay

Total protein extracts were prepared from NRK-52E cells as previously described [5]. Protein was measured using Bradford protein assay (Pierce, Rockford, IL, USA). Bovine serum albumin was used as the standard. DNase activity was determined using SRED assay in total protein extracts [42]. For that, Difco DNase Test Agar (BD, Sparks, MD, USA) was reconstituted in water (4.2% *w*/*v*), autoclaved, and distributed to 100 mm Petri dishes. Series of 3 mm holes were prepared in cold agar and 5 μL of cell lysate were applied to each hole for 16 h at 37 °C. Remaining DNA was visualized by precipitation with 1M HCl followed by staining with ethidium bromide, and imaged with the Eagle Eye imaging system. Human recombinant DNase I (Genentech) was used as the positive control. Densitometric analyses were done by using Image J 1.30v, in which the mean optical density was calculated per a fixed area in the circle around each well.

### 4.6. RNA and DNA Extraction

Total RNA from NRK-52E cells or rat brain nuclei was extracted using RNeasy Mini Kit (QIAGEN, MD, USA) followed by DNA removal using RNase-free DNase kit (QIAGEN GmbH, Hilden, Germany). DNA was extracted from cultured NRK-52E cells using DNeasy blood and tissue kit (QIAGEN).

### 4.7. PCR and Real-Time RT-PCR

PCR was done using extracted DNA from the NRK52E cells in a 25-µL reaction using SmartCycler (Cepheid, Sunnyvale, CA, USA). Reaction mix was prepared using Advantage^®^-GC2 PCR kit (Clontech, Mountain View, CA, USA) according to manufacturer recommendations. All primers for the endonucleases and their annealing temperatures are shown in Table S1. PCR products were separated in 1% agarose gel, stained with ethidium bromide and photographed under UV light in BIO-RAD imaging system (Hercules, CA, USA). For real time RT-PCR, the previously described protocol was followed [34]. Briefly, total RNA (5 µg) was reverse-transcribed in a 50-µL reaction followed by real-time RT-PCR in a 25-µL reaction using SmartCycler (Cepheid). Reaction mix was prepared using Platinum SYBR Green qPCR Supermix-UDG (Invitrogen) according to manufacturer recommendations. Two-temperature cycles with annealing/extension were used. The fluorescence was measured at the end of the annealing step. The melting curve analyses were performed at the end of the reaction (after 45th cycle) between 60 °C and 95 °C to assess the quality of the final PCR products. The threshold cycles, C(t), values were calculated by fixing the basal fluorescence at 15 units. The standard curve of the reaction effectiveness was performed using the serially diluted (5 points) mixture of all experimental cDNA samples in triplicates for each endonuclease and 18 s RNA separately. Calculation of the relative RNA concentration was performed using Cepheid SmartCycle software (Version 2.0d). Data are presented as the ratio of endonuclease/18 s mRNA.

### 4.8. Western Blotting

Protein was separated in an 11.5% gel according to the Laemmli’s procedure [43]. The total protein extract from cells (10–100 µg) was dissolved in 50 mM Tris-HCl, pH 6.8, 1% SDS, 2 mM EDTA, 1% 2-mercaptoethanol and 7.5% glycerol, and denatured by heating at 100 °C for 10 min. Electrophoresis was performed at 100 V for 2 h. Proteins were transferred onto the nitrocellulose membrane in Novex transferring buffer (Invitrogen) at 40 V for 3 h. The membranes were stained with Ponseau S (Sigma, St. Louis, MO, USA) to control the equal protein load as described elsewhere [44]. After soaking in the blocking solution overnight at 4 °C, the membrane was incubated with polyclonal anti-EndoG antibody (Millipore, Billerica, MA, USA) diluted 1:1000, polyclonal Anti-CAD antibody (Abbiotec, San Diego, CA, USA) diluted 1:500, polyclonal anti-GAPDH (Abcam, Cambridge, MA, USA) antibody diluted 1:1000, or anti-DNase γ, anti-DNase 2β, anti-DNase X and DNase I like 2 antibodies (Santa Cruz Biotechnology, Santa Cruz, CA, USA) diluted 1:500, then washed in Tris-buffered saline (TBS), pH 7.6. Primary antibodies, including anti-EndoG, anti-CAD and anti-GAPDH were detected with anti-rabbit IgG-horseradish peroxidase (HRP) while anti-DNase γ, anti-DNase 2β, anti-DNase X and DNase I like 2 were detected with donkey anti-goat IgG-horseradish peroxidase (HRP) using SuperSignal chemiluminescent kit (Pierce Biotechnology, Rockford, IL, USA).

### 4.9. Immunocytochemistry and Image Analyses

Cells were fixed with 5% formalin and the immunostaining was performed as described previously [45]. The cells were immunostained with rabbit anti-EndoG at 1:200 dilution (Millipore) or anti-CAD at 1:100 dilution (Abbiotec) in dilution buffer (0.5% BSA, 0.05% Tween-20, phosphate saline buffered (PBS)). The primary antibodies were detected with 1:400 diluted anti-rabbit IgG-AlexaFluor 594 conjugates (Invitrogen). Control sections were performed by substituting the primary antibody with dilution buffer only. Cells were mounted under cover slips with Prolong^®^ Antifade kit (Invitrogen, Carlsbad, CA, USA) and acquired using the Olympus IX-51 inverted microscope (Olympus America, Center Valley, PA, USA) equipped with an ORCA-ER monochrome camera (Hamamatsu Photonics K.K., Hamamatsu City, Japan). CFP and AlexaFluor 594 were visualized using the following settings for excitation/emission: 438/483 and 562/624 respectively. Image analysis was performed using SlideBook 6.2 software. For quantification, 10 independent fields of view were collected per each rat kidney section, and mean optical density (MOD) was recorded for AlexaFluor 594 channel. The data were presented as averages of MOD_x_/field of view for each channel.

### 4.10. Statistics

Statistical analysis was performed with 2-way ANOVA and Student’s *t*-test. Results were expressed as mean ± standard error of mean (SEM). *p* < 0.05 was considered significant.

## Supplementary Materials

Supplementary Materials can be found at https://www.mdpi.com/1422-0067/21/22/8665/s1.
