# Supplementary material for: DNase I Induces Other Endonucleases in Kidney Tubular Epithelial Cells by Its DNA-Degrading Activity
Source: Int J Mol Sci. 2020 Nov 17;21(22):8665. doi: 10.3390/ijms21228665 (PMC7698339; doi:10.3390/ijms21228665)
Supplement: Supplementary file 1 [file ijms-21-08665-s001.pdf]

SUPPLEMENT

TABLES AND FIGURES

DNase I induces other endonucleases in kidney tubular epithelial cells by its DNA-degrading activity

Tariq Fahmi [1], Xiaoying Wang [1], Dmitry D. Zhdanov [1], Intisar Islam [1],

Eugene O. Apostolov [1], Alena Savenka [1], and Alexei G. Basnakian [1,2]

[1] *Department of Pharmacology & Toxicology, University of Arkansas for Medical Sciences, 4301 West Markham Street, #638, Little Rock, AR 72205*

[2] *Central Arkansas Veterans Healthcare System, 4300 West 7th Street, Little Rock, AR 72205*

TABLE S1. **Primers used for PCR and real-time RT-PCR**

Primers for real time RT-PCR				
**Target**	**Sense (5′-3′)**	**Antisense (5′-3′)**	**Product size, bp**	**Annealing temp.,°C**
**DNase I**	**TGCTGTTGGGAAGCTACTGGATGA**	**TCTCGGCTGAAGGTGTCATTTCCA**	**198**	**65**
**DNase X**	**GCATACATAGCAATGCGTGGCCTT**	**TCAGTGTCAGTCTGTGGGCATTGA**	**109**	**64**
**DNase I like 2**	**AGACCTAAGTGCAGTGTCCTTGCT**	**ATACTGGTCACGTCCAAGTGGCTT**	**100**	**65**
**DNase ϒ**	**TCGGCCAGCTTTATAAGTCAGCGA**	**TGGGTACAGGGACATCTTTGCACT**	**85**	**65**
**DNase 2α**	**AGTTTCCAGGGATTGGCAAGCAAC**	**ATTTGGCAAAGCTCTGGAAGGTGG**	**195**	**64**
**DNase 2β**	**AAGTGGCCTCTGCATCACTCTCAA**	**TGTGGATGAGTTCCCAGCGAAAGA**	**125**	**65**
**L-DNase 2**	**TGCCCGAAGACATTGAGGATGAGT**	**CACGTTTGGTCCATTCACGCAGTT**	**90**	**64**
**CAD**	**AGGACCCATCCTGGTTTGAAGGTT**	**TGTACTGCACGGACTTGAGCTTGT**	**198**	**64**
**EndoG**	**GCATGCCTGGAACAACCTTGAGAA**	**TGCCTCCAGGATCAACACCTTGAA**	**193**	**64**
**18s**	**GGATCCATTGGAGGGCAAGT**	**ACGAGCTTTTTAACTGCAGCAA**	**91**	**64**
**EndoG (mouse)**	**GAATGCCTGGAACAACCTTGAGAG**	**TGCTCCAGGATCAGCACCTTGAA**	**193**	**63**
**β-actin**	**TGAGAGGGAAATCGTGCGTGACAT**	**ACCGCTCATTGCCGATAGTGATGA**	**152**	**65**

Primers for PCR				
**Target**	**Sense (5′-3′)**	**Antisense (5′-3′)**	**Product size, bp**	**Annealing temp.,°C**
**DNase X**	**TTGGCTGAGGCAAGCGTCGG**	**GCCAGGTCCTCATCAGGCCG**	**2949**	**59**
**DNase I like 2**	**GCTTTTGCCCTGATCCTCAGTTAATGG**	**TGAACATTAAGATCAAGCTCTGCAGCC**	**2473**	**59**
**DNase ϒ**	**AAAGTGGCCCAGAGGGATCCAGTAATT**	**CCTGGGACTATGCCCTTTTGAGAATCCA**	**2903**	**60**
**DNase 2α**	**AGACCCAGGTCCCTGAAGTCTTTAG**	**TTCCCCTTGAGACAGTGTTTCTGTGTTG**	**2586**	**56.5**
**DNase 2β**	**GCATCAGATGACATGAGACTCAGCCTC**	**TCTTCAGCAACACCCCTCAGCTATTTC**	**2997**	**58**
**L-DNase 2**	**CAGCTAAGCAAGAGGTAAGGTCCT**	**TTCTGGGATCCAAACTCAGCTTCTC**	**3064**	**57.5**
**CAD**	**TGGGCTTGAATGGGCGCTGG**	**TAGACGTGTACCGCCCGCCA**	**2510**	**59**
**EndoG**	**TACGGGCCGGTTTGACCCTGG**	**TGGGAACTATGTATGGTGGGGACC**	**3039**	**56**
**GAPDH**	**CTCTCTGCTCCTCCCTGTTCTAGAGACA**	**ATGGCATGGACTGTGGTCTAGAAAGCAT**	**3008**	**61**
**Rpl13a**	**AAGATGGCGGAGGGGCAGGTTC**	**AGAGTCCATTGGTCTTGAGGACCTCTG**	**2646**	**58**
**Rplp0**	**CCCCTTTTCGGAACTAGCTCACTCAC**	**CAAATTAAGCAGGCTGACTTGGTGTGAG**	**2741**	**58**

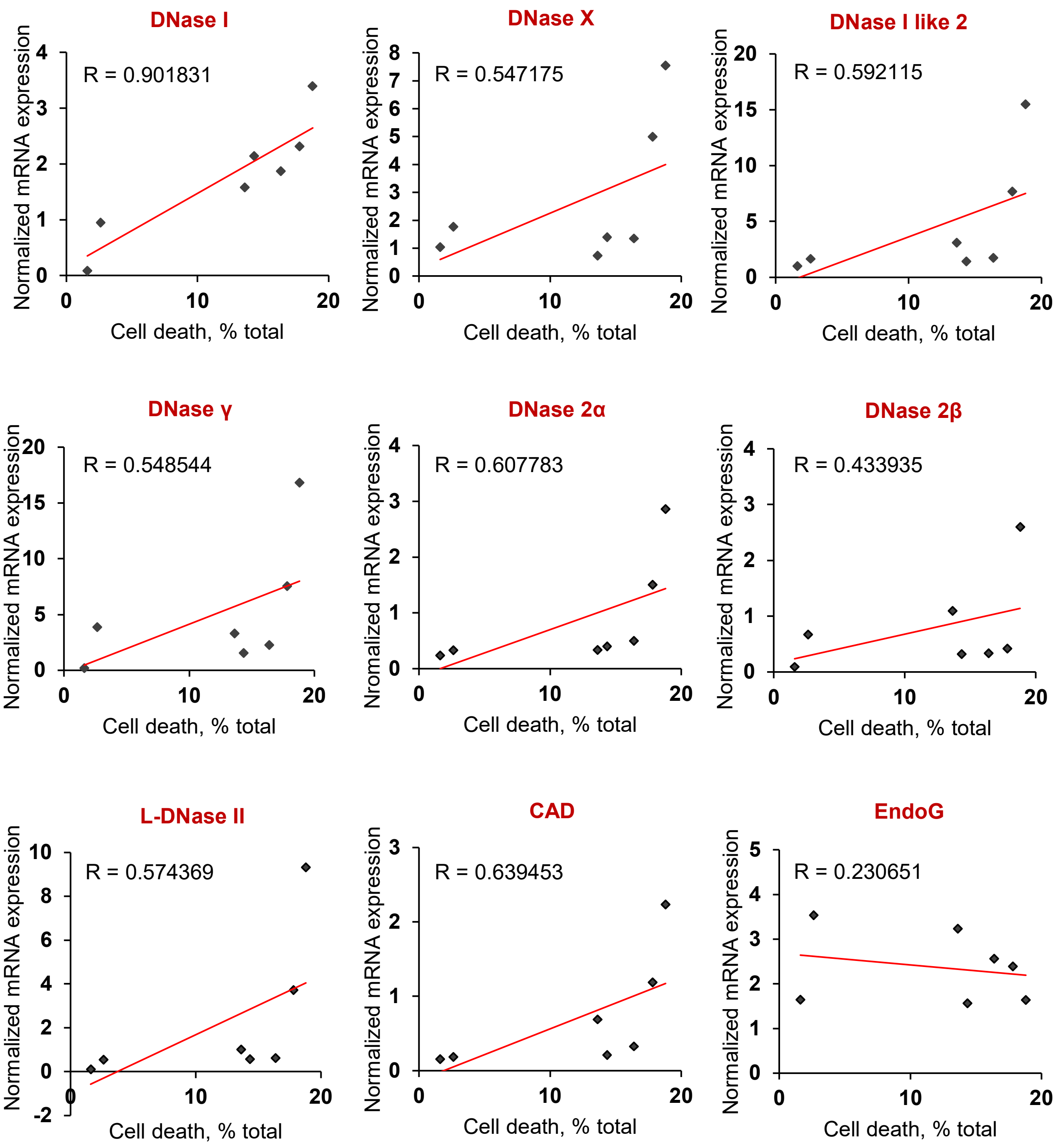

Supplement Figure S1. Correlation of endonucleases' expression with cell death. Correlation between mRNA expression of each endonuclease measured by real time RT-PCR and cell death measured by PI assay at varying time points. All endonuclease except EndoG show positive correlation.
